# mRNA-specific readthrough of nonsense codons by antisense oligonucleotides (R-ASOs)

**DOI:** 10.1093/nar/gkae624

**Published:** 2024-07-16

**Authors:** Denis Susorov, Dimas Echeverria, Anastasia Khvorova, Andrei A Korostelev

**Affiliations:** RNA Therapeutics Institute, UMass Chan Medical School, 368 Plantation Street, Worcester, MA 01605, USA; RNA Therapeutics Institute, UMass Chan Medical School, 368 Plantation Street, Worcester, MA 01605, USA; RNA Therapeutics Institute, UMass Chan Medical School, 368 Plantation Street, Worcester, MA 01605, USA; RNA Therapeutics Institute, UMass Chan Medical School, 368 Plantation Street, Worcester, MA 01605, USA

## Abstract

Nonsense mutations account for >10% of human genetic disorders, including cystic fibrosis, Alagille syndrome, and Duchenne muscular dystrophy. A nonsense mutation results in the expression of a truncated protein, and therapeutic strategies aim to restore full-length protein expression. Most strategies under development, including small-molecule aminoglycosides, suppressor tRNAs, or the targeted degradation of termination factors, lack mRNA target selectivity and may poorly differentiate between nonsense and normal stop codons, resulting in off-target translation errors. Here, we demonstrate that antisense oligonucleotides can stimulate readthrough of disease-causing nonsense codons, resulting in high yields of full-length protein in mammalian cellular lysate. Readthrough efficiency depends on the sequence context near the stop codon and on the precise targeting position of an oligonucleotide, whose interaction with mRNA inhibits peptide release to promote readthrough. Readthrough-inducing antisense oligonucleotides (R-ASOs) enhance the potency of non-specific readthrough agents, including aminoglycoside G418 and suppressor tRNA, enabling a path toward target-specific readthrough of nonsense mutations in *CFTR*, *JAG1*, *DMD*, *BRCA1* and other mutant genes. Finally, through systematic chemical engineering, we identify heavily modified fully functional R-ASO variants, enabling future therapeutic development.

## Graphical Abstract

**Graphical Abstract:**
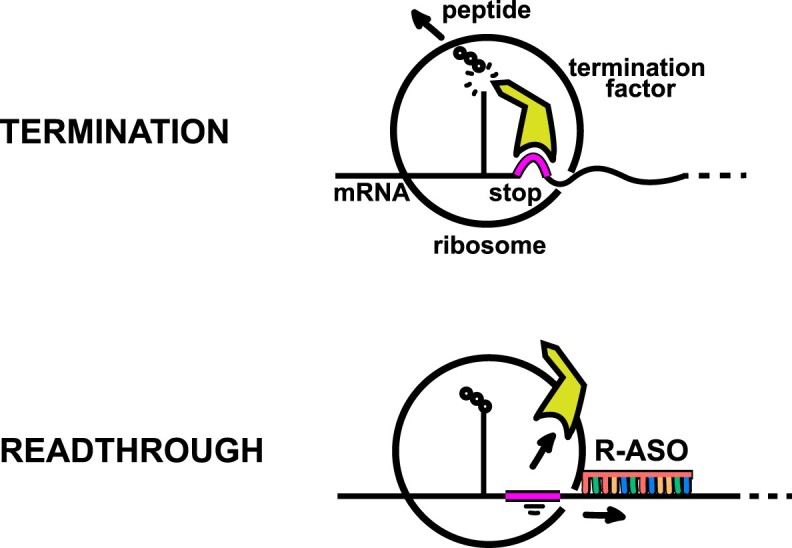

## Introduction

Nonsense mutations underlie more than 10% of human genetic disorders, including cystic fibrosis, Alagille syndrome, Duchenne muscular dystrophy, Rett syndrome, and hereditary cancers (1), which lack effective treatments. Nonsense mutations—sense codons mutated to stop codons—cause premature translation termination, the production of truncated proteins, and nonsense-mediated mRNA decay, resulting in loss- or gain-of-function phenotypes. Restoring full-length translation is the primary goal of treating nonsense-associated diseases. Nevertheless, even partial restoration of full-length product may provide significant therapeutic benefit (2).

Whereas sense codons are recognized and decoded by aminoacyl-tRNAs (aa-tRNA), stop codons—UAA, UAG and UGA—are recognized by release factor eRF1 in eukaryotes. Upon stop-codon recognition, eRF1 catalyzes the irreversible hydrolysis of the peptidyl-tRNA ester linkage, releasing the peptide from the ribosome. For a readthrough to occur, a nonsense codon must be read by an aa-tRNA rather than by eRF1, so that an amino acid is incorporated, allowing translation to continue to the normal stop codon at the end of the open reading frame. The readthrough of nonsense codons can be induced by enhancing stop-codon decoding by an aa-tRNA, inhibiting termination, or both. For example, a suppressor tRNA is a modified tRNA whose anticodon binds to a stop codon and outcompetes eRF1 (3–6). Whereas aminoglycoside drugs (e.g. G418) are thought to both induce miscoding and inhibit termination (7–9), other small-molecules downregulate or inhibit eRF1 (10–12), inhibit nonsense-mediated decay of the mRNA (13,14), or act via less understood mechanisms, (e.g. ataluren) (15–17). None of these approaches specify the nonsense mRNA and may cause miscoding or readthrough of authentic stop codons, yielding aberrant proteins (3,8,18).

Structural and biochemical studies have revealed a key distinction between decoding and termination. Whereas aa-tRNA recognizes a codon by forming three base pairs in the decoding center, eRF1 interacts with the stop codon and with the nucleotide immediately after the stop codon, as if ‘pulling’ the mRNA into the entry tunnel (19–21). Indeed, a longer mRNA fragment is protected by the ribosome during termination than during aa-tRNA decoding (22,23). We hypothesize that interfering with mRNA dynamics at the entry tunnel might therefore inhibit termination and stimulate nonsense readthrough.

Antisense oligonucleotides (ASOs) are a powerful tool to modulate gene expression, due to their high specificity achieved by Watson–Crick base pairing (24). ASO-based therapies promise to treat many genetic diseases (25), and several ASO drugs have been approved by the FDA (24,26). ASOs act via several molecular mechanisms, including RNA interference, RNase H-mediated cleavage, splicing modulation, non-coding RNA inhibition, gene activation, and programmed gene editing (24,26). Here, we present a new mode of action that enables antisense oligonucleotides to bind a specific position downstream of the stop codon to induce readthrough. These readthrough-inducing antisense oligonucleotides, or R-ASOs, can restore translation of nonsense-containing mRNAs in a mammalian translation system to levels that might be therapeutically relevant if achieved in patients. Using a recently developed kinetic assay (7), we show that R-ASOs inhibit translation termination in a context-specific manner, consistent with the hypothesized mechanism of action. Cooperativity between R-ASOs and other readthrough inducers—G418 or suppressor tRNA—suggests a way to induce readthrough at specific disease-causing nonsense codons. The conceptual demonstration of this new mechanism of nonsense readthrough is an important step toward mRNA-specific therapies against hundreds of severe genetic diseases.

## Materials and methods

### Oligonucleotides

Oligonucleotides were ordered from IDT, Gene Link or Gene Tools, or they were synthesized in house, as described (27).

### Preparation of mRNAs and ser-tRNA^UGA^

#### mRNAs

DNA constructs coding for model mRNAs were ordered from Genewiz (Azenta) (Figure 2A shows the principal design of these constructs). Point mutations were introduced using the Q5® Site-Directed Mutagenesis Kit (NEB) or by PCR with overlapping primers. All PCR reactions were performed with Phusion® High-Fidelity DNA Polymerase (NEB), and all constructs were sequenced by Genewiz (Azenta).

DNA templates for *in vitro* transcription were generated by PCR using primers annealing to globin UTRs: the forward 5′-tttttTAATACGACTCACTATAGACACTTGCTTTTGACACAACTGTG-3′ (IDT) primer containing a T7-promoter (underscored) and the reverse 5′-ttttttttttttttttttttttttttttttGCAATGAAAATAAATTTCCTTTATTAGCC-3′ (IDT) primer coding for a 30-nt poly-A-stretch.

The PCR reactions were extracted with phenol:chloroform (pH 8.0), and the amplified transcription templates were precipitated from the aqueous fraction with 0.3 M NaOAc and ethanol. The DNA pellets were washed with cold 80% ethanol, dissolved in mQ (deionized) water and used for *in vitro* transcription.

*In vitro* transcription reactions containing DNA template, 4 mM each rNTP, 5% homemade T7-polymerase (7), TBx1 (166 mM Hepes–KOH, pH 7.5, 20 mM Mg(OAc)_2_, 40 mM DTT, 2 mM spermidine, 100 μg/μl BSA (NEB)), were incubated at 37°C for 3 h. After incubation, the magnesium pyrophosphate precipitate was removed by centrifugation and RNA was precipitated from the supernatant with 2.5 M LiCl. The pellet was washed with cold 80% ethanol and dissolved in mQ water.

To test whether mRNA capping affects R-ASO-induced readthrough, we compared translation reactions using capped and uncapped mRNAs in rabbit reticulocyte lysates (RRL). mRNAs were capped using the Vaccinia Capping System (NEB); 30-nucleotide poly(A) tails were further elongated by the *Escherichia coli* Poly(A) Polymerase (NEB), according to the manufacturer's protocol.

*In vitro* RNase H assays were performed with *E. coli* RNase H (NEB) according to the manufacturer′s protocol.

The integrity of *in vitro* transcribed mRNA was assessed by electrophoresis in TBE–1% agarose gels, and mRNA concentration was determined using a Nanodrop spectrophotometer (Thermo Scientific).

#### Ser-tRNA^UGA^

The sequence of human tRNA^Ser^ was retrieved from the tRNA-database (28,29) (tRNA-Ser-CGA-1-1, http://gtrnadb.ucsc.edu/index.html). A DNA plasmid encoding tRNA-Ser-CGA-1-1 with its CGA anticodon sequence changed to UCA (to match the UGA stop codon) was ordered from Genewiz (Azenta). This plasmid was used to amplify a tRNA^UGA^*in vitro* transcription template by PCR with the following primers (synthesized by IDT): a forward 5′-TTTTTTAATACGACTCACTATAGCTGTGATGGC-3′ primer containing the T7-promoter (underscored) and a reverse 5′-mTmGGCGCTGTGAGCAGGATTCG-3′ (IDT) primer, containing 2′-OMe-modified nucleotides (underscored) to preclude T7-polymerase from adding non-template nucleotides to the 3′-CCA end of tRNA^UGA^ transcription products (30,31). This PCR product was used as a template for *in vitro* transcription, as described above. The transcription reaction was extracted with phenol:chloroform (pH 4.5), and the transcription products were precipitated from the aqueous fraction with 0.3 M NaOAc and ethanol. The nucleic acid pellet was washed with cold 80% ethanol and dissolved in mQ water. Free nucleotides were removed by repeated ultrafiltration using Amicon Ultra 10-kDa-cutoff centrifugal filters (MilliporeSigma). The concentration of tRNA^UGA^ was determined using a Nanodrop spectrophotometer (Thermo Scientific).

The tRNA^UGA^ products were aminoacylated in a reaction containing 2.5 mM tRNA^UGA^, 6 mM ATP, 0.05 mM serine, 5% yeast S100 extract, 1 mM DTT, and ABx1 (50 mM Hepes–KOH, pH 7.5, 30mM KCl, 50 mM MgCl_2_, 30 μM MnCl_2_, 30 μM ZnCl_2_). Yeast S100 extract was prepared as described (32). The aminoacylation reaction was incubated in a thermocycler (Bio-Rad) at 25°C for 25 hours. The aminoacylated products were extracted and precipitated, and the extent of aminoacylation was assessed by fractionation on an 8 M urea, 7% polyacrylamide gel. The gel was stained with 0.2% methylene blue and imaged using a ChemiDoc imager (Bio-Rad).

### Translation in rabbit reticulocyte lysates

*In vitro* translation was performed as described (7). A reaction mixture containing 50% rabbit reticulocyte lysate (RRL, nuclease-treated; Promega), 30 mM Hepes–KOH (pH 7.5), 50 mM KOAc, 1.0 mM Mg(OAc)_2_, 0.2 mM ATP, 0.2 mM GTP, 0.04 mM of 20 amino acids (Promega), 2mM DTT, and 1% furimazine (Promega) nanoluciferase substrate. The reaction mixture was aliquoted 9 μl per well into 384-well plates (Corning Low Volume White Round Bottom). Water, ASO (10 μM final), G418 (0.5 μM final), or Ser-tRNA^UGA^ (0.5 μM final) were added to the mixture and incubated 5 min at 30°C in a microplate reader (Tecan INFINITE M1000 PRO or Tecan Spark). Translation reactions were initiated by adding mRNA (30 nM final) and luminescence signal was recorded for 20 min in kinetic mode. Reactions were stopped by transferring to 100 μl of TRIzol reagent (Invitrogen), and total protein and total RNA fractions were extracted according to the manufacturer's instructions for further analyses.

Changes in luminescence signal over time were used to derive rates of translation in relative luciferase units per second (RLU/s). Maximal values were used to compare reaction conditions. Translation rates were calculated in Microsoft Excel and plotted in GraphPad Prism version 9.4.1.

### Western blotting of the total protein fraction

Protein pellets from TRIzol extractions were dissolved in 20 μl of 80 mM Tris–HCl (pH 6.8), 2% SDS, 20% glycerol, 10% β-mercaptoethanol, 8 M urea with repeated vortexing and heating at 95°C for 10 min. The denatured samples were fractionated on discontinuous polyacrylamide gels (5% concentrating gel, 12% resolving gel) containing 8 M urea. Proteins were electrophoretically transferred to PVDF membrane using a Trans-Blot SD semi-dry transfer cell (BioRad). Membranes were blocked for 1 h at room temperature in PBS containing 0.1% Tween 20 (PBST) and 5% nonfat dry milk (ChemCruz) and then incubated overnight at 4°C in PBST containing GT517 monoclonal antibody against the Strep tag (1:500, Invitrogen). Membranes were washed and incubated for 1 h at room temperature in PBST containing HRP-linked goat anti-mouse secondary antibody (1:2000, Invitrogen). Membranes were at last washed, incubated with HRP-detection reagent (SuperSignal West Atto kit; Thermo Scientific), and imaged using a ChemiDoc sytem (BioRad) set for automatic exposure.

### mRNA detection in the total RNA fraction

To assess the integrity of model mRNAs after translation, we developed an approach based on agarose electrophoresis in the presence of fluorescent oligonucleotide probes. Total RNA pellets from TRIzol extractions were dissolved in 40 mM PIPES (pH 6.8), 100 mM NaCl, 1 mM EDTA, 90% deionized formamide, and 1 μM each of three FAM-labeled DNA oligonucleotides (IDT)—one annealing to a CFTR sequence, and two annealing to nanoluciferase sequences. Samples were heated for 2 min at 95°C, then placed on ice and mixed with loading buffer (40 mM PIPES, pH 6.8, 6% glycerol, 5 mM EDTA, 12.5% deionized formamide, 0.025% bromophenol blue). Samples were fractionated through 2% agarose in TBE. The gels were cut just above bromophenol band (to remove non-bound FAM-labeled probes) and imaged using a ChemiDoc (BioRad) in ‘epi/fluorescein’ mode. Gels were then stained with SYBR Safe DNA stain (Invitrogen) and imaged using a ChemiDoc (BioRad) to visualize the total RNA in ‘SYBR Safe’ mode.

### Termi-Luc assay

The Termi-Luc assay was performed as described (7), with modifications. A 1 ml translation reaction was assembled in RRL (as above) supplemented with 1.2 μM mutant human eRF1^AAQ^ and incubated at room temperature for 5 min. Model mRNAs (24 μg) were added to reaction mixtures and incubated for 10 min at 30°C. Translation reactions were stopped by adjusting Mg(OAc)_2_ to 5 mM and KOAc to 300 mM. The reactions were then centrifuged through 10%–35% sucrose gradients (Beckman Coulter ultracentrifuge, SW41 Ti rotor), and gradients were fractionated using a gradient master and piston fractionator (Biocomp). The peak A_254_ fractions containing 80S ribosomes were collected and concentrated to 3.5 *A*_260_ U/μl by ultrafiltration using Amicon 50-kDa cutoff centrifugal filters (MilliporeSigma) in 50 mM Hepes–KOH, pH 7.5, 100 mM KCl, 5mM Mg(OAc)_2_, 5% glycerol, 2 mM DTT.

10 μl aliquots of ribosomal complexes were premixed with 158 μl of buffer DBx1 50 mM Tris–HCl, pH 7.5, 100 mM KCl, 2.5 mM Mg(OAc)_2_, 0.014% Triton X-100 to get 12 reaction mixtures with the volume of 14 μl. The inclusion of detergent (Triton X-100) significantly increased the reproducibility and signal level of the assay compared to our original procedure without detergent (7). Substituting Tween 20 or BSA for Triton X-100 also improved the assay. The aliquots were applied to the 384-well plate (Corning Low Volume White Round Bottom), mixed with oligonucleotides (to 10 μM) or mQ water, and incubated in the multiplate reader for 5 min at 30°C. Furimazine (Promega) was added to 1% and the baseline signal was recorded for 2 min. Then yeast eRF1*eRF3*GTP complex (prepared as in (7)) and preincubated in buffer DBx1, supplemented with 0.2 mM GTP) was added to the reaction (to 0.05 μM) to the final volume of 20 μl and luciferase signal was recorded in kinetic mode.

GraphPad Prism version 9.4.1 was used to fit the raw curves to the exponential plateau equation and to plot *k*_obs_ values.

### Bioinformatics

To generate semi-random sequences for the +8 to +27 site from a redundant RY-denoted template (R-purines, Y-pyrimidines), a Bash script was written, randomly generating an A or G for purine residues and a T or C for pyrimidine residues. Three resulting sequences were ordered from Genewiz (Azenta).

To isolate +8 to +27 contexts in reported human nonsense mutation sites, transcripts with nonsense mutations and the positions of the premature stop codons were retrieved from the LOVD3 database (33) (https://databases.lovd.nl/shared/genes). Premature stop codons arising from frameshift mutations were excluded. Coding sequences corresponding to nonsense transcripts were retrieved as FASTA sequences using NCBI Batch Entrez (https://www.ncbi.nlm.nih.gov/sites/batchentrez). The FASTA sequences and premature stop positions were used to create a CSV database of premature stops contexts with the help of a Bash script. The database was queried using awk/grep commands.

### Miscellaneous

All calculations and data visualization were done in Microsoft Excel and GraphPad Prism version 9.4.1. Statistical analyses were performed in GraphPad Prism 10.2.3 (403) using recommended options. Figures were prepared in Adobe Illustrator. Ribosome thumbnails were prepared with the help of ChimeraX (34,35) and Adobe Illustrator using the structural model of the rabbit 80S ribosome (PDB: 6MTB). The schematic image of Ser-tRNA^UGA^ was prepared with Forna at RNAfold server (http://rna.tbi.univie.ac.at//cgi-bin/RNAWebSuite/RNAfold.cgi) using secondary structure constraints retrieved along with the tRNA-Ser-CGA-1-1 sequence from the tRNA-database (http://gtrnadb.ucsc.edu/index.html). Graphic alignment of +8 to +27 contexts was performed using WebLogo (36) (https://weblogo.berkeley.edu/logo.cgi). The pictures of modified nucleotides were prepared in ChemSketch version 2022.1.0. Gel quantifications were done in ImageJ 1.54g (37).

## Results

### R-ASO-induced readthrough of stop codons depends on the targeting position downstream of the stop codon

**Figure 1.**
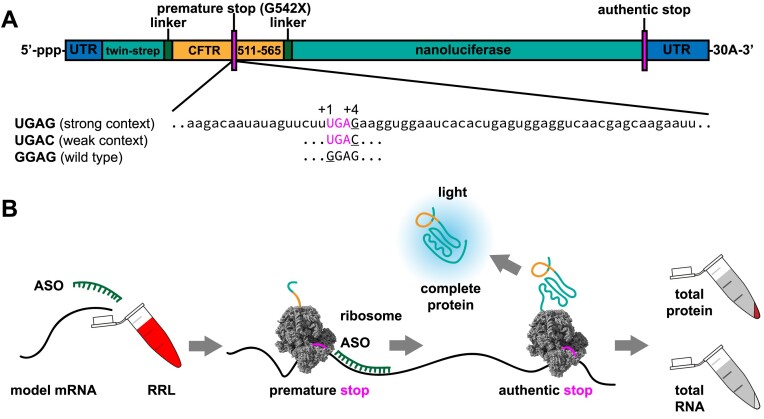
Experimental system to measure translation efficiency of stop-codon readthrough using a nanolucifease reporter as a full-protein readout in rabbit reticulocyte lysate. **(A)** The scheme for model mRNAs used for in vitro translation reactions. (**B**) The scheme of in vitro translation experiments used in this work.

To study the ability of antisense oligonucleotides to induce readthrough of premature stop codons, we first developed a reporter mRNA in which translation of nanoluciferase requires the readthrough of the CFTR G542X mutation, the most frequent nonsense mutation in cystic fibrosis patients (Figure 1A). In the *CFTR* mRNA, the G542X nonsense codon is immediately followed by a guanosine nucleotide (i.e. UGAG). A stop codon followed by a +4 purine (where +1 defines the first nucleotide of the stop codon) comprises a ‘strong’ termination site: the +4 purine forms stabilizing interactions with 18S rRNA in eRF1-bound ribosomes (19,21). By contrast, termination can be 10-fold less efficient at a stop codon with a +4 pyrimidine (8,38–41). We therefore also generated a reporter with a ‘weak’ G542X nonsense codon followed by a +4 cytosine (i.e. UGAC) to readily detect readthrough. To control for maximal CFTR::nanoluciferase expression, we generated a reporter mRNA with the wild-type CFTR G542 sense codon (i.e. GGAG) in frame with nanoluciferase. We then estimated the rate of translation of each reporter mRNA in rabbit reticulocyte lysates by recording luminescence in real time (Figure 1B, Supplementary Figure S1).

**Figure 2.**
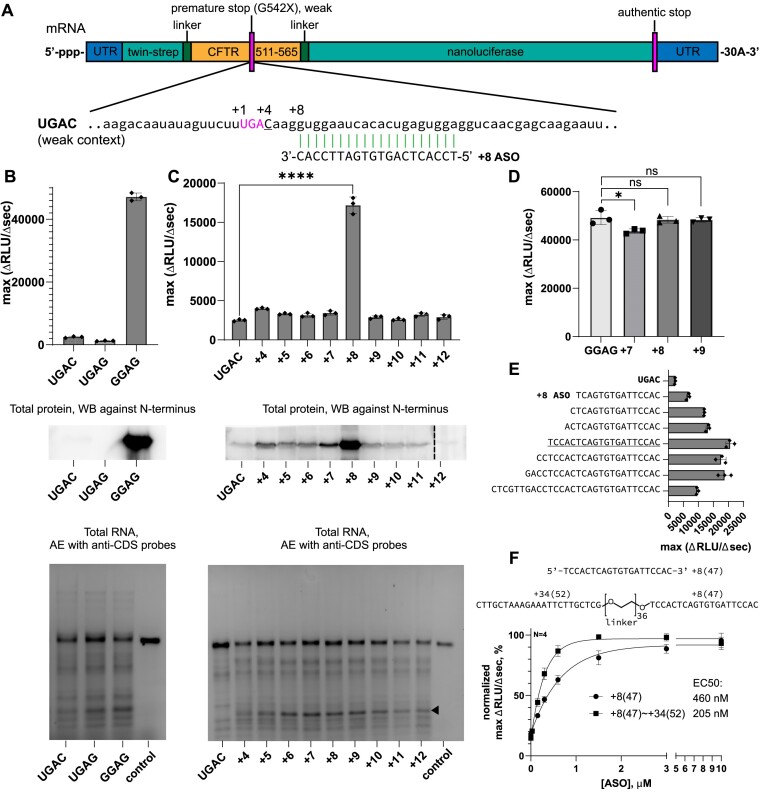
Oligonucleotides induce sequence-specific readthrough of nonsense codons. (**A**) Example of a model reporter mRNA encoding a nonsense-containing fragment of CFTR (G542X mutant) upstream of nanoluciferase. (**B**) Upper: Translation efficiencies of model mRNAs encoding the nonsense codons ^+1^UGAC^+4^ and ^+1^UGAG^+4^ are substantially lower than that of the control encoding the sense codon GGA (mean ± s.d., *n* = 3). Middle: Western blot analysis of full-length protein product (via Strep-Tag antibody). Lower: RNA gel showing mRNA levels after the translation reaction as detected by agarose electrophoresis with fluorescent probes complementary to CFTR and nanoluciferase coding sequences. The black arrow marks an mRNA degradation product observed with DNA ASOs. The ‘control’ lane shows full-length mRNA before translation in RRL. (**C**) Translation efficiencies of nonsense-containing mRNA with oligonucleotides targeting different positions downstream of the nonsense codon ^+1^UGAC^+4^ (mean ± s.d., *n* = 3; ****P < 0.0001 according to one-way ANOVA followed by Dunnett's test for multiple comparisons); the full-length protein and mRNA levels are shown in lower panels as described in (B). The dashed line separates 2 different gels that were processed in parallel and visualized at similar exposure times. (**D**) Bar graph showing the translation efficiency of the wild-type (sense codon) CFTR reporter in the absence or presence of +7 to +9 ASOs (mean ± s.d., *n* = 3; ns, non-significant; *P < 0.5 according to one-way ANOVA followed by Dunnett's test for multiple comparisons). (**E**) Bar graph showing the effect of +8 ASO length on readthrough efficiency. The 20-mer +8 R-ASO was used in most experiments unless otherwise stated (underlined). (**F**) Upper: Schematics showing the sequence structure of the individual +8 R-ASO 20-mer and the ‘double-oligo’ with +8 R-ASO conjugated to an oligonucleotide targeting position +34 downstream of the stop codon. Lower: Plots showing readthrough efficiencies as a function of +8 R-ASO or double-oligo concentration; half maximal effective concentrations (EC50) are reported (mean ± s.d., *n* = 4). RLU, relative light units; AE, agarose electrophoresis; WB, western blotting; CDS, coding sequence.

Wild-type CFTR G542 (GGAG) reporter mRNA was efficiently translated, producing an abundance of full-length CFTR::nanoluciferase fusion protein confirmed by western blot (Figure 2B, Supplementary Figure S2A). By contrast, the nonsense-containing mRNAs were translated ∼20-fold less efficiently than the wild-type reporter mRNA (Figure 2B). The levels of protein detected by western blotting correlated with the translation efficiencies measured in the luminescence assay (Figure 2B, Supplementary Figure S2A). We observed similar levels of nonsense-containing and wild-type mRNAs at the end of the assay, indicating that reduced protein output did not result from mRNA degradation (Figure 2B, Supplementary Figure S2A, B).

To test the hypothesis that oligonucleotides induce stop-codon readthrough, we designed a series of DNA oligonucleotides (∼20-nt, with similar predicted Tm) complementary to the *CFTR* mRNA, each starting at a different position (+4 to + 12) downstream of the stop codon (Figures 2A, C). We first analyzed readthrough in the context of the UGAC nonsense codon, which is expected to be more susceptible to readthrough. Most oligonucleotides only slightly (<1.5-fold) enhanced the readthrough of UGAC over background (Figure 2C). The outlier was the +8 ASO, which increased the readthrough signal 6.8-fold over background (Figure 2C), consistent with the higher protein production (Figure 2C, middle panel, Supplementary Figure S2C). Importantly, the ASOs did not affect translation of the wild-type reporter mRNA, indicating that double-helical structures do not significantly block ribosomal translocation along mRNA (Figure 2D). Varying the length of the +8 ASO revealed that 20 nucleotides of complementarity most efficiently induced readthrough (Figure 2E). The same stimulatory effects were observed for mRNAs bearing a 5′ cap and a longer poly(A)-tail, characteristic features of cellular mRNAs (Supplementary Figure S3A). Stimulation of readthrough was dose-dependent: readthrough was stimulated by as little as 150 nM +8 ASO (5-fold excess over mRNA), with half maximal efficiency (EC50) achieved at 460 nM +8 ASO (Figure 2F). To determine if the efficiency of the +8 ASO could be improved by increasing its affinity, we conjugated the +8 ASO to an oligonucleotide that anneals further downstream (starting at +34; Figure 2F). The double-oligo conjugate improved the efficiency of readthrough, yielding an EC50 of 205 nM (Figure 2F). These results indicate that the +8 ASO induces efficient and specific readthrough of a nonsense mRNA without modifying the expression of the wild-type mRNA. We termed this ASO a +8 readthrough-inducing antisense oligonucleotide, or +8 R-ASO.

Analysis of mRNA integrity revealed an additional mRNA fragment after incubation with DNA ASOs in RRL, suggesting partial mRNA degradation (Figure 2C, lower panel, black arrow, Supplementary Figure S2C). The mRNA might be cleaved by RNase H, whose activity in RRL was previously reported (42). Notably, the level of DNA-induced fragmentation of mRNA in the RRL did not correlate with readthrough efficiency (Figure 2C; also see below), indicating that degradation does not substantially interfere with the readthrough mechanism under these reaction conditions. Indeed, translation was potently inhibited by pretreating mRNAs with +8 R-ASO and *E. coli* RNase H (Supplementary Figure S4A) or by adding *E. coli* RNase H to translation reactions in the presence of +8 R-ASO (Supplementary Figure S4B). These results argue against a role for RNase H in the readthrough mechanism.

### R-ASO synergize with G418 or suppressor tRNA to induce readthrough

**Figure 3.**
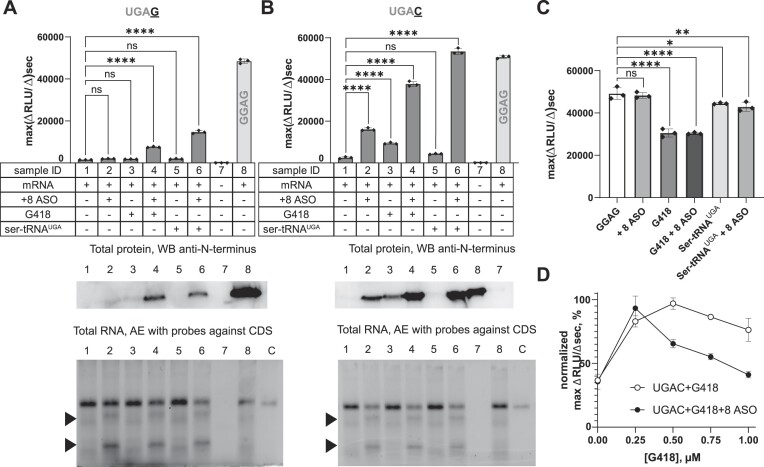
Enhancement of R-ASO-induced readthrough by G418 and suppressor tRNA. (A, B) Upper: Bar charts showing the translation efficiencies of reporters with UGAG (**A**) and UGAC (**B**) stop codons and the effects of combinations of readthrough inducers, +8 ASO, G418, or Ser-tRNA^UGA^. (mean ± s.d., *n* = 3; ns, non-significant; *P< 0.5; ****P< 0.0001; significance determined by one-way ANOVA followed by Dunnett's test for multiple comparisons). Middle: Western blots of full-length protein product (via Strep-Tag antibody). Lower: RNA gels showing mRNA levels after the translation reactions, as in Figure 2B. Lane C shows the intact mRNA before translation. Arrows mark additional mRNA degradation products observed with DNA ASOs. (**C**) Bar graphs showing the effects of readthrough inducers on translation of wild-type (nonsense-free) mRNA sequence (mean ± s.d., *n* = 3; ns, non-significant; *P< 0.5; ****P< 0.0001; significance determined by one-way ANOVA followed by Dunnett's test for multiple comparisons). (**D**) Plot showing the effect of G418 dose on UGAC readthrough in the presence or absence of +8 R-ASO (mean ± s.d., *n* = 2).

Whereas the +8 R-ASO strongly induced readthrough on the weak UGAC nonsense codon, it only slightly induced readthrough on the authentic CFTR G542X UGAG nonsense codon (Figure 3A, Supplementary Figure S2D). This result suggests that the +8 R-ASO does not efficiently reduce eRF1 binding and termination at a strong stop codon. Because combinations of readthrough-promoting molecules can synergistically increase readthrough efficiency (11,15,43,44), we asked if the +8 R-ASO can synergize with G418 or an artificial UGA-suppressor tRNA (Ser-tRNA^UGA^) (Supplementary Figure S5) to enhance readthrough. In titration experiments, maximal readthrough of the UGAC construct was achieved with 0.5 μM G418 (Figure 3D). We therefore used 0.5 μM G418 or 0.5 μM Ser-tRNA^UGA^ in subsequent synergy experiments. Alone, G418 or Ser-tRNA^UGA^ partially stimulated readthrough of the weak *CFTR* UGAC nonsense site (3.8-fold and ∼1.5-fold, respectively), but not of the strong *CFTR* UGAG nonsense site (Figure 3B and A, Supplementary Figure S2D). The +8 R-ASO synergized with G418 or Ser-tRNA^UGA^ to enhance readthrough of both weak and strong nonsense sites (Figure 3B and A, Supplementary Figure S2D), exceeding the sum of individual effects of readthrough compounds. Synergy was most pronounced for the weak UGAC site (15- to 20-fold increase), producing near wild-type levels of the reporter protein (Figure 3B). On the UGAG nonsense site, synergy between +8 R-ASO and G418 (5-fold) or Ser-tRNA^UGA^ (10-fold) restored the reporter protein to ∼25% of the level produced by the wild-type *CFTR* reporter (Figure 3A).

In addition to testing the effect of the +4 context on +8 R-ASO-induced readthrough efficiency, we tested the stop codon identity, which reportedly affects readthrough in the following manner: UGA > UAG > UAA (45). Replacing the stop codon in the UGAC model mRNA with a UAG or UAA stop codon, we observed a similar order of readthrough efficiency (Supplementary Figure S3B). Accordingly, the combination of +8 R-ASO and G-418 increased the readthrough of the strong UAG and UAA stop codons to a lesser degree than of the UGA stop codon (Supplementary Figure S3B).

Aminoglycosides are toxic *in vivo* at concentrations that induce nonsense readthrough (46). Indeed, in a control experiment, G418 inhibited translation of the wild-type-like GGAG mRNA, in keeping with its indiscriminate miscoding effects (Figure 3C). In G418 dose-response experiments, maximum readthrough was achieved with half as much G418 in the presence of +8 R-ASO than in its absence (Figure 3D). Combinatorial therapies might therefore reduce the non-specific readthrough and toxic side effects of G418.

### R-ASOs inhibit translation termination

**Figure 4.**
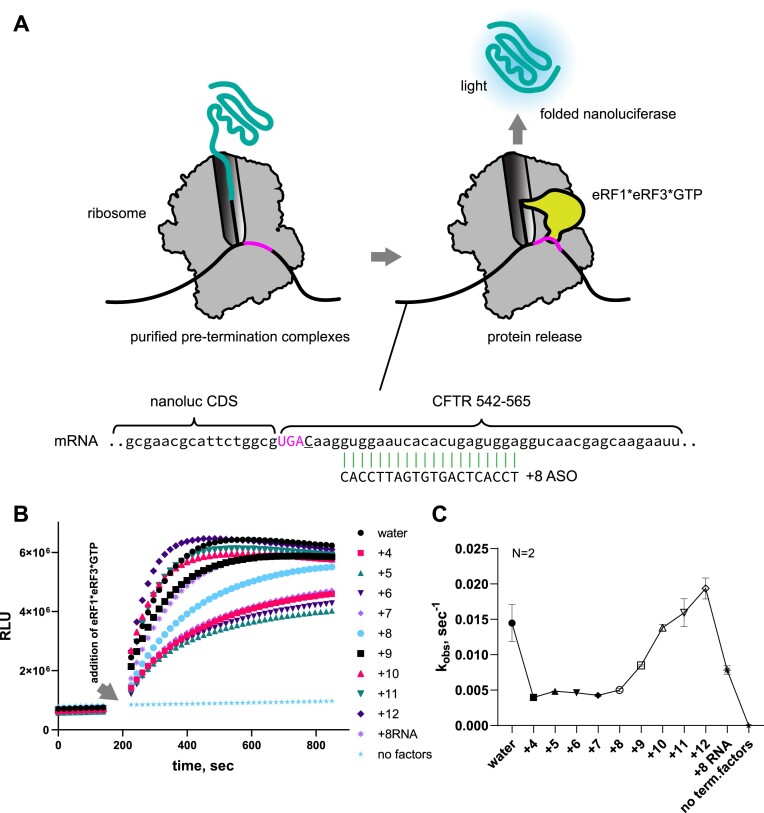
Dependence of translation termination on DNA oligonucleotides annealing downstream the stop codon. (**A**) Schematic of the Termi-Luc assay to measure the kinetics of nanoluciferase release from purified pre-termination complexes. (**B**) Plots showing the effects of oligonucleotides on nanoluciferase luminescence over time after the addition of termination complex. (**C**) Plot showing the effect of oligonucleotide position on the release rate (mean ± s.d., *n* = 2).

To test whether the ASOs affect translation termination, we used a recently developed assay to measure the kinetics of full-protein release from pre-termination complexes (7). We purified 80S ribosomes paused at the weak CFTR UGAC nonsense site with nascent nanoluciferase attached to the P-site tRNA (Figure 4A). Addition of the ternary eRF1*eRF3*GTP termination complex releases nanoluciferase from the ribosome, increasing the luminescence signal (Figure 4B) (7). Preincubation of 80S complex with oligonucleotides resulted in varying termination rates. Termination was significantly inhibited by ASOs annealing at positions +4 through +8, partially inhibited by the ASO at position +9, and unaffected by ASOs at positions +10 through +12 (Figure 4C). These results suggest that the inhibition of termination depends on the proximity of the oligonucleotide-mRNA duplex to the A site or ribosomal mRNA entry tunnel. Since only the +8 R-ASO promotes nonsense readthrough in the complete RRL system (Figure 2C), we hypothesize that the translating ribosome displaces oligonucleotides that anneal closer to the A site (+4 to +7), thus preventing them from inhibiting termination (also see Discussion). In summary, the ability of the +8 R-ASO to induce stop-codon readthrough is at least in part due to the inhibition of termination at the specific stop codon.

### Modified R-ASOs retain readthrough activity and reduce mRNA degradation

Our initial experiments using cost-effective DNA oligonucleotides revealed minor degradation of mRNA (Figure 2C, Supplementary Figure S2C) due to a low activity of RNase H in rabbit reticulocyte lysates (42). In cells or *in vivo*, however, DNA oligonucleotides readily induce RNase H-mediated cleavage of the complementary RNAs (47). Such activity may interfere with the attempts to promote readthrough of nonsense-containing mRNAs *in vivo*. Moreover, unmodified oligonucleotides are highly unstable in cells and must be modified to induce long-lasting therapeutic effects *in vivo* (26,48). We therefore tested the effects of R-ASO nucleotide modifications on readthrough efficiency and mRNA stability.

**Figure 5.**
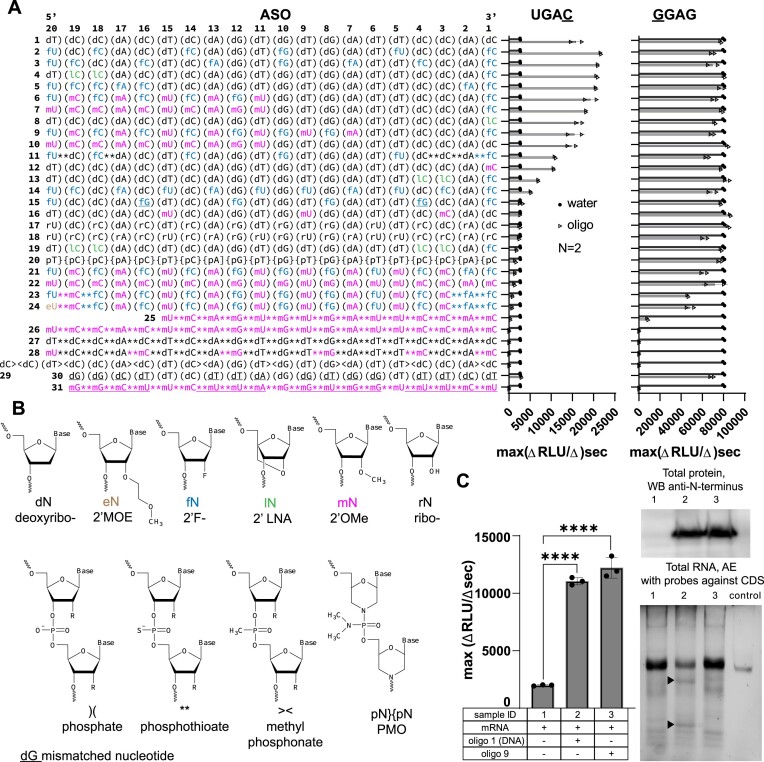
Nucleotide modifications affect readthrough efficiency of R-ASOs. (**A**) Plots showing the readthrough efficiency on UGAC and translation efficiency on GGAG reporters in the presence of the indicated modified +8 R-ASOs (mean ± s.d.; *n* = 2). (**B**) Chemical structures of the modifications tested in (A). (**C**) Left: Bar graph showing the effects of the original DNA +8 R-ASO or the modified +8 R-ASO (#9 in panel A) on UGAC CFTR mRNA translation efficiency on the (mean ± s.d., *n* = 3; ****P< 0.0001; significance was determined by one-way ANOVA followed by Dunnett's test for multiple comparisons). Upper right: Western blots of full-length protein product after the translation reaction (via Strep-Tag antibody). Lower right: RNA gels showing mRNA levels after the translation reaction, as in Figure 2B.

We designed and tested a panel of +8 R-ASOs with a variety of nucleotide modifications used in therapeutic oligonucleotides (Figure 5A and B) (26). Using the weak UGAC reporter we found that readthrough activity depended on the positions and types of nucleic acid modifications. In general, oligonucleotides with modifications toward the 5′ end retained activity (Figure 5A, oligo 9, 10; positions 20–7). Compound 9 had fourteen modifications at 5′ positions and retained most of the +8 R-ASO activity. By contrast, the 3′ end was sensitive to modifications. Whereas 2′-locked nucleic acid (LNA) or 2′-fluoro (compounds 2–9, 11) modifications at the 3′-terminal nucleotide were well tolerated, a 2′-OMe modification significantly reduced readthrough activity (Figure 5A). Extensive 2′-Fluoro modification of the 3′ end also reduced readthrough efficiency (Figure 5A, oligo 14). As expected, mismatched DNA, DNA/2′F oligonucleotides were inactive, consistent with the target-specific readthrough mechanism (Figure 5A, oligos 15, 30).

Phosphorothioate (PS) linkages also reduced readthrough activity depending on the number of substitutions (Figure 5A, compare oligos 2 and 11). Fully PS-modified +8 ASOs completely inhibited translation from both UGAC and GGAG mRNAs (Figure 5A, oligos 25, 28). The inhibitory effect of phosphorothioate on translation was not sequence-specific, as the mismatched PS oligo also inhibited translation (Figure 5B, oligo 31), consistent with the non-specific toxicity of highly PS-modified oligonucleotides *in vivo* (49).

Together these results reveal that activation of readthrough strongly depends on the positions of modifications in the 5′ and 3′ ends of +8 R-ASOs. Whereas the 5′ end can be heavily modified without compromising readthrough, the 3′ end—likely positioned at the mRNA entrance to interact with the ribosome—is sensitive to the types and positions of modified nucleotides. Through this analysis, we have identified an active readthrough-promoting R-ASO with only five unmodified DNA nucleotides in the 20-mer (Figure 5A, oligo 9). Importantly, +8 R-ASO #9 substantially reduced mRNA degradation (Figure 5C), in keeping with the reduced activity of RNase H in the presence of modified oligonucleotides (Supplementary Figure S4C). Thus, +8 R-ASO #9 represents a scaffold for the future development of R-ASOs for *in vivo* applications.

### +8 mRNA sequence context affects the readthrough by R-ASOs

**Figure 6.**
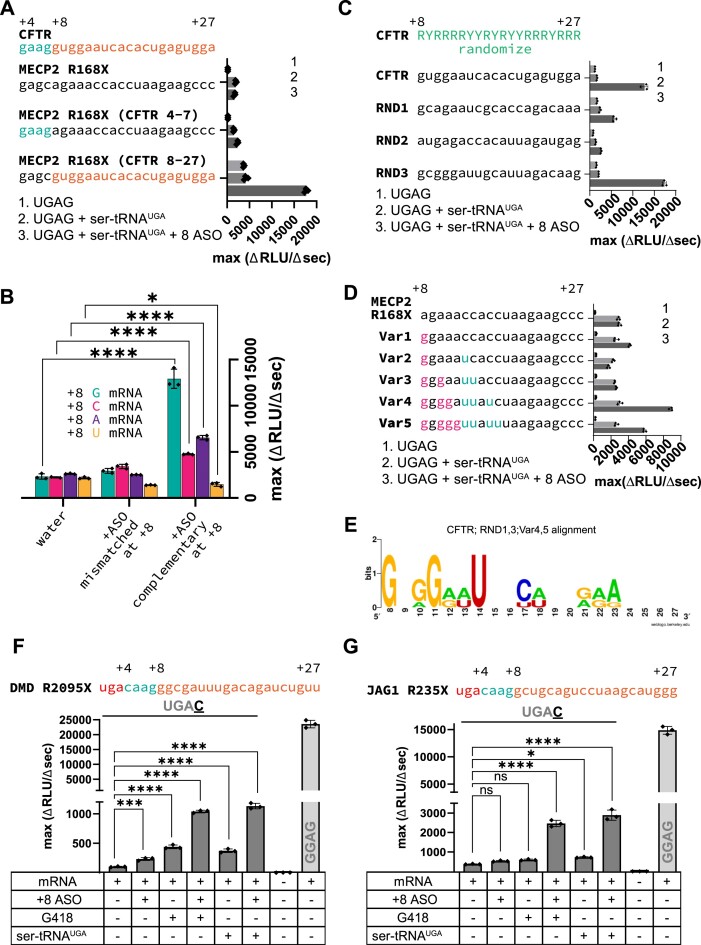
+8 mRNA context affects the readthrough by R-ASOs, enabling nonsense readthrough in DMD and JAG1 genes. (**A**) Readthrough efficiencies of UGAG *MECP2* constructs carrying local substitutions with *CFTR* sequences (mean ± s.d., *n* = 3). (**B**) Readthrough efficiencies of UGAC*CFTR* constructs carrying +8 substitutions (mean ± s.d., *n* = 3; *P< 0.5, ****P< 0.0001 determined by to two-way ANOVA followed by Sidak's test for multiple comparisons). (**C**) Readthrough efficiencies of three UGAG CFTR-like sequences randomized at positions +8 to +27 (mean ± s.d., *n* = 3). (**D**) Readthrough efficiencies of UGAG *MECP2* constructs with substitutions in the +8 to +27 region (mean ± s.d., *n* = 3). (**E**) Sequence alignment of *CFTR*; RND 1,3; Var 4, 5, +8 to +27 contexts. (**F**) Complementary +8 R-ASO and its combinations with G418 or Ser-tRNA^UGA^ induce readthrough on *DMD* R2095X reporter mRNA (mean ± s.d., *n* = 3; ***P< 0.001, ****P< 0.0001 determined by one-way ANOVA followed by Dunnett's test for multiple comparisons). (**G**) Complementary +8 R-ASO and its combinations with G418 or Ser-tRNA^UGA^ induce readthrough on a *JAG1* R253X reporter mRNA (mean ± s.d., *n* = 3; ns, non-significant, *P< 0.5, ****P< 0.0001 determined by one-way ANOVA followed by Dunnett's test for multiple comparisons).

To test whether ASOs can stimulate readthrough of another disease-causing premature stop, we designed a reporter mRNA encoding the Rett syndrome *MECP2* nonsense mutation R168X (Figure 6A, Supplementary Figure S6A). The R168X nonsense codon is immediately followed by a +4 G nucleotide, making it a strong stop codon. Nevertheless, a +8 ASO failed to induce readthrough in the presence or absence of G418 or Ser-tRNA^UGA^ (Figure 6A, Supplementary Figure S6A). Previous studies showed that mRNA nucleotides downstream of the stop codon (including position +8) can affect termination and readthrough (38,50,51). To identify additional determinants of mRNA*R-ASO activity, we substituted two regions of *MECP2* downstream of the nonsense codon with sequences from *CFTR*. In one construct, we replaced nucleotides + 4 to +7 (i.e. immediately after the nonsense codon), and in the second, we replaced nucleotides +8 to +27 (i.e. the +8 ASO-binding site; Figure 6A). The latter construct substantially improved the readthrough activity of the *MECP2* nonsense reporter (4.7-fold, Figure 6A).

We next sought to identify sequence properties in the R-ASO-binding region of the CFTR mRNA that confer readthrough sensitivity. We first mutated the +8 position, which appeared most important in the register-shifting (Figure 2C) and R-ASO modification experiments (Figure 5A). We modified the weak *CFTR* nonsense reporter mRNA, substituting +8 G with A, C, or U, and tested the ability of complementary or mismatched +8 ASOs to induce readthrough (Figure 6B). Readthrough was only stimulated if +8 ASOs base pair at the +8 position of the nonsense site (Figure 6B). Furthermore, readthrough efficiency was much higher with +8 G than any other nucleotide (Figure 6B). These results indicate that ASO-induced readthrough requires base-pair interactions near the mRNA tunnel and correlate with the importance of the +8-nucleotide identity for the readthrough efficiency in mammalian cells (38).

To further dissect mRNA-based determinants of readthrough, we randomized purine and pyrimidine nucleotides in the +8 to +27 region of CFTR mRNA (RYRRRRYYRYRYYRRRYRRRR, where R denotes purines and Y pyrimidines), while retaining similar predicted stability (Tm) of the corresponding duplexes. We then substituted these sequences in the corresponding region of the *CFTR* nonsense reporter and measured the ability of complementary +8 ASOs to induce readthrough (Figure 6C). Of the three semi-random sequences (RND1–3), RND2 was nearly inactive, RND1 was slightly less active than the original CFTR sequence, and RND3 demonstrated higher readthrough efficiency than CFTR (Figure 6C). The inactive RND2 reporter had a +8 G-to-A replacement, resembling the inhibitory effect of the +8 substitutions in the CFTR mRNA (Figure 6B). Most notably, we observed a preference for G nucleotides at +8 and nearby positions in readthrough-prone sequences (CFTR, RND1 and RND3).

These analyses suggest that the context of the *MECP2* R168X mutation—with an A at position +8 and only one guanosine among the five initial positions (Figure 6A, D)—prevented ASO-induced readthrough. To test this hypothesis, we made five variant *MECP2* nonsense reporters, each with a +8 G and sequential G nucleotides added near the +8 position. We preserved the overall G/C-content by converting downstream C nucleotides to U nucleotides (Figure 6D). A single +8 G substitution enabled R-ASO-induced readthrough in the presence of Ser-tRNA^UGA^ (∼2-fold relative to tRNA alone). ASO-induced readthrough was most efficient on a variant reporter with four sequential G nucleotides from +8 to +11 (∼3-fold greater than tRNA alone; Figure 6D). These findings indicate that R-ASO-induced readthrough depends on the identity of mRNA nucleotides in the R-ASO-binding region, suggesting that disease-causing nonsense mRNAs with guanosines at +8 and neighboring positions are most likely to respond to R-ASO treatments.

### +8 R-ASO allows readthrough of disease-causing nonsense mutations in DMD, JAG1 and BRCA1, but not in HBB

To evaluate the extent of nonsense mutations that might be prone to ASO-induced readthrough, we compiled a table of +8 to +27 sequences from human premature stop codons annotated in Leiden Open Variation Database 3.0 (33,52). Using the consensus sequence obtained from our most active readthrough constructs as a query (Figure 6E), we identified 197 nonsense mutations in 181 human genes that might be prone to +8 ASO-induced readthrough. The LOVD3 database reports 176 of the 197 nonsense mutations as pathogenic or likely pathogenic. Of the 48 examples with a C nucleotide at +4 position, we have tested three nonsense contexts in our model mRNAs. The most abundant variant in this list, the R2095X mutation (UGA) in the Duchenne muscular dystrophy *DMD* gene (53,54) demonstrated readthrough in response to +8 R-ASO combined with G418 or Ser-tRNA^UGA^ (Figure 6F). A model construct for the *BRCA1* Q1785X mutation associated with breast cancer (55) contains a UAG premature stop codon followed by an R-ASO-sensitive context (+4C; +8G, +11G). As expected, readthrough was induced by the combination of +8 R-ASO and G418 (Supplementary Figure S6C). Readthrough on a construct encoding the *JAG1* R235X mutation causing Alagille syndrome (56) was also stimulated in the presence of +8 R-ASO and G418 or Ser-tRNA^UGA^ (Figure 6G). JAG1 R235X deviates slightly from our initial consensus (i.e. with a G instead of U at +14), indicating that a broader set of contexts may be susceptible to R-ASOs. Using only +8 G as a criterion (Figure 6B), we identified 3983 nonsense mutations contexts (17.8% of all sequences in the database) in 2065 unique human genes, representing a large selection of diseases potentially amenable to R-ASO-induced readthrough.

While our work was underway, Kar *et al.* reported ASO-induced readthrough of an *HBB* W16X nonsense reporter in HEK293 cells (57). Using a DNA vector for the model protein expression, the authors reported nonsense readthrough by DNA oligonucleotides annealing at +4 and + 12 positions (the authors term these positions +1 and +9 relative to the nucleotide following the stop codon). They reported nearly complete restoration of full-length protein product, despite the particularly strong stop-codon context (UAGG). We therefore independently evaluated their findings in our system (Supplementary Figure S6B). Readthrough was neither induced by the oligonucleotide sequences reported in the original study (57) nor by a complementary +8 ASO, either alone or combined with G418 (Supplementary Figure S6B). The lack of readthrough is consistent with the strong stop codon and suboptimal sequence downstream of the stop codon (e.g. +8 A). In sum, Kar *et al.* findings contrast the R-ASO principles identified in our work, where neither the +4 nor the +12 oligonucleotides induce the readthrough (Figure C), and the readthrough of the strong stop codon is low in the absence of additional readthrough inducers (Figure 3A, Supplementary Figure S3B). We emphasize that the features of termination observed in our lysate system closely replicate those observed *in vivo* and numerous cellular studies (8,39,45), including the dependence of readthrough efficiency on the stop-codon type and on the +4 nucleotide (Figure 3A, B, Supplementary Figure S3B). These results further highlight the strong mechanistic support for the R-ASO-induced readthrough of stop codons depending on mRNA sequence contexts.

## Discussion

**Figure 7.**
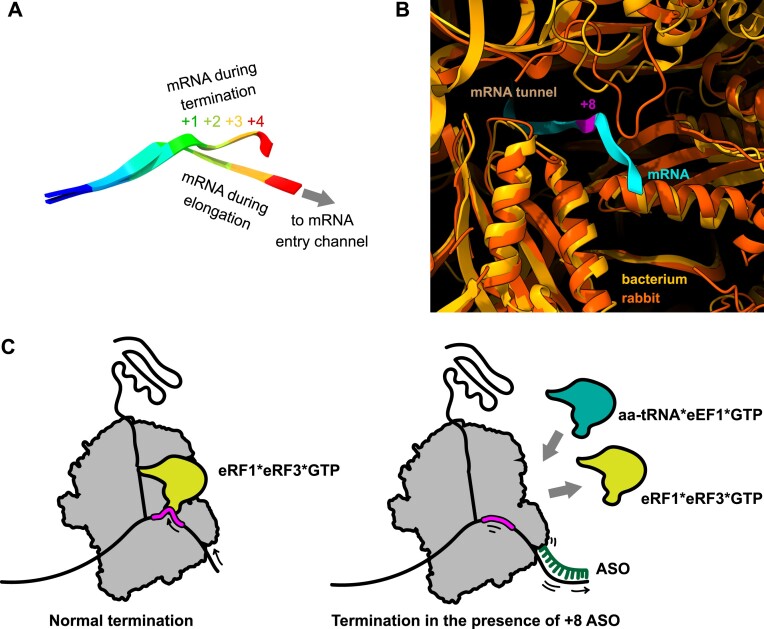
Mechanistic model for R-ASO induced readthrough of premature stop-codons. (**A**) Comparison of mRNA positions in the ribosomal A site during elongation and termination. PDB codes: 5LZS and 5LZT, respectively. (**B**) Comparison of mRNA entry channels of bacterial (yellow) and mammalian (orange) ribosomes. PDB codes: 6BY1 (ribosome B) and 5LZS, respectively. The structures were superimposed by ribosomal proteins underlining the entry channel (S3, S4, S5). (**C**) Proposed mechanism for nonsense readthrough induced by R-ASOs binding downstream of the stop codon.

Our results demonstrate that ASOs that target downstream of a premature stop codon can induce readthrough and thus enable mRNA-sequence-specific nonsense suppression. The readthrough efficiency depends on the position and nucleotide identities of the R-ASO*mRNA duplex. In several nonsense mutation contexts, including disease-causing *CFTR* G542X, *DMD* R2095X, *JAG1* R235X and *BRCA1* Q1785X, oligonucleotides binding at the +8 position induced efficient readthrough. Interaction between the R-ASO*mRNA duplex and the ribosomal mRNA entry channel likely induces readthrough by preventing the mRNA from being pulled into the A site for stop-codon recognition by eRF1 (19,21) (Figure 7A, C). Indeed, structural and biochemical studies identified the +8 position at the entry tunnel of the bacterial ribosome as the first mRNA nucleotide available for interaction with a complementary oligonucleotide (58–60) (Note: the authors call this position +11 counting from the first nucleotide of the P-site codon (59)). The mRNA tunnels of the bacterial and mammalian ribosomes are structurally similar (Figure 7B). The +8 position may therefore be the first available point where the R-ASO*mRNA duplex can sterically block termination. This idea is supported by our kinetic measurements of protein release, showing that duplexes at the +8 position strongly inhibit termination, whereas those that bind farther from the entry tunnel fail to inhibit termination (Figure 4C). mRNA secondary structures downstream of a nonsense codon also promote readthrough and have been proposed to do so by sterically inhibiting termination (50,61).

ASO-induced readthrough is more effective on a weak UGAC nonsense codon context than on a strong UGAG, consistent with the importance of the +4 nucleotide in stop-codon termination and readthrough (8,38,39). R-ASOs nevertheless induce readthrough on strong stop codons in the presence of G418 or suppressor tRNA, which alone are inefficient (Figure 3A). These results highlight the fact that stop codon readthrough is a competitive process in which termination and elongation work against each other. Thus, dampening termination down with R-ASO while fueling elongation up with G418 or a suppressor tRNA allows for efficient stop codon readthrough. Interestingly, aminoglycosides were shown to promote miscoding and, at higher concentrations, inhibit termination (7,9). Future experiments are required to determine if R-ASOs, in addition to inhibiting termination, can enhance binding of suppressor or near-cognate tRNAs to stop codons (Figure 7C).

Whereas ASOs annealing at position +4 to +8 all inhibited termination on purified pre-termination complexes (Figure 4C), only the +8 R-ASO promoted readthrough in RRL (Figure 2C). This is likely because in the termination assay, the +4 to +7 ASOs displace the mRNA from its normal position in the ribosomal tunnel, which is too narrow to accommodate the mRNA*ASO duplex close to the A site, and they thus prevent eRF1 interaction with the stop codon. In the translation extract, however, the translocating ribosome must easily unwind duplexes at +4 to +7 positions (58–60), until encountering the stop codon. Indeed, efficient helicase activity of elongating ribosomes in RRL is supported by the fact that complementary ASOs do not affect expression of the control wild-type CFTR-construct (GGAG, Figure 2D). The pausing at the stop codon likely allows the oligonucleotide at +8 to remain bound at the mRNA entrance tunnel and prevent the ribosome from properly reading the stop codon (Figure 7C).

Our findings reveal that the +8 position strongly affects the sensitivity to readthrough. At the +8 position, guanosine is most favorable for readthrough, followed by A, and then by either pyrimidine (Figure 6B). The preference for G might reflect its ability to stabilize a duplex (62,63), but a +8 C does not induce readthrough, suggesting that specific interactions between the +8 R-ASO*mRNA duplex and the ribosome also play a role. An increased GC-content downstream of the +8 nucleotide also improves readthrough (Figure 6D). While the local duplex stability appears to be an important factor of readthrough efficiency, additional effects, such as interactions with the ribosome components, may be involved. The other very important outcome is that the readthrough effect is highly specific to the sequence downstream of the stop codon of interest (Figures 2B, 5A and 6B), allowing R-ASOs to avoid off-target readthrough.

Whereas the 5′ end of +8 R-ASO tolerates chemical modifications, the 3′ end–which anneals near the ribosomal mRNA entry tunnel–is sensitive to modifications (Figure 5A). This asymmetry is consistent with the idea above that the mechanism of readthrough depends on interactions between the +8 R-ASO*mRNA duplex and the mRNA entry tunnel. RNA-DNA duplexes have unique properties that distinguish them from RNA or DNA duplexes (64). In particular, RNA-DNA duplexes can adopt intermediate A/B-form helix conformations that might facilitate the installation of an R-ASO at the mRNA entry channel to inhibit translation termination at the upstream nonsense codon.

In summary, this work demonstrates the fundamental feasibility of R-ASO-induced readthrough of nonsense codons in an mRNA-specific manner and establishes that nucleotide contexts in both the mRNA and R-ASO are important for readthrough. Based on our analyses of nonsense codon and mRNA contexts, we estimate that 17% of disease-causing nonsense mutations—underlying hundreds of genetic disorders—could be relevant R-ASO targets. ASO-induced readthrough may be less toxic compared to molecules that promote non-specific readthrough. Although the cellular lysate used in this work recapitulates many aspects of cellular translation (65), future studies in cellular and animals models are needed to translate the approach into therapeutics to treat devastating genetic diseases.

## Supplementary Material

gkae624_Supplemental_File

## Data Availability

All study data are included in the article and/or supporting information. Plasmids are available upon request.
