# Supplementary material for: mRNA-specific readthrough of nonsense codons by antisense oligonucleotides (R-ASOs)
Source: Nucleic Acids Res. 2024 Jul 16;52(15):8687–701. doi: 10.1093/nar/gkae624 (PMC11347175; doi:10.1093/nar/gkae624)
Supplement: gkae624_Supplemental_File [file gkae624_supplemental_file.pdf]

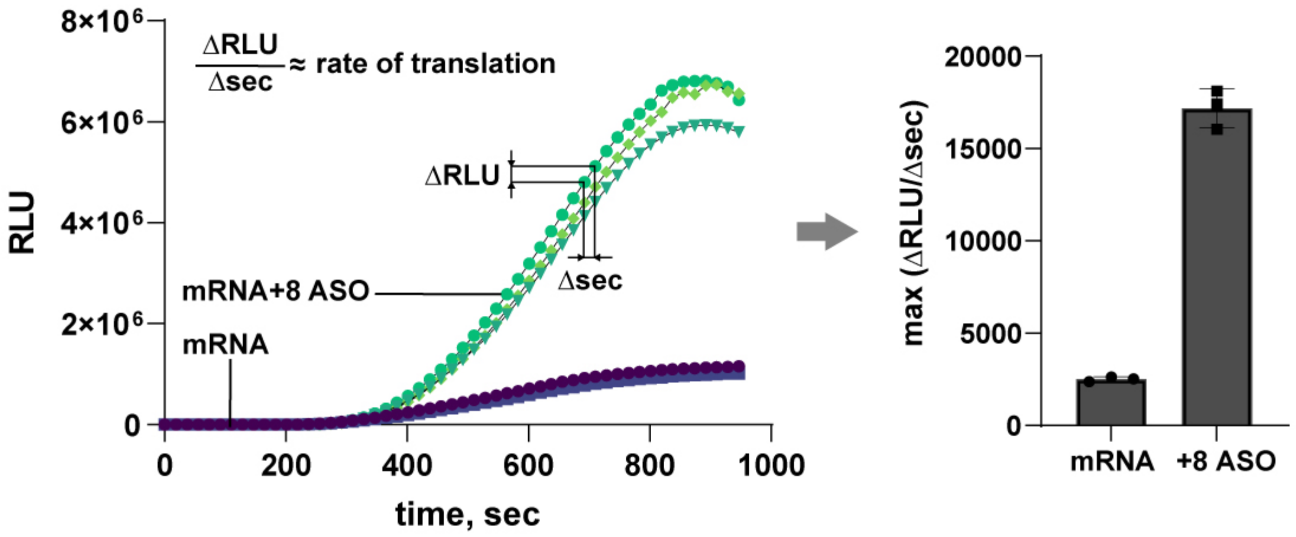

Figure S1. The scheme for calculating translation rates from time-progress luminescence curves. Partial data from Figure 2C are shown as an example. RLU-relative luminescence units.

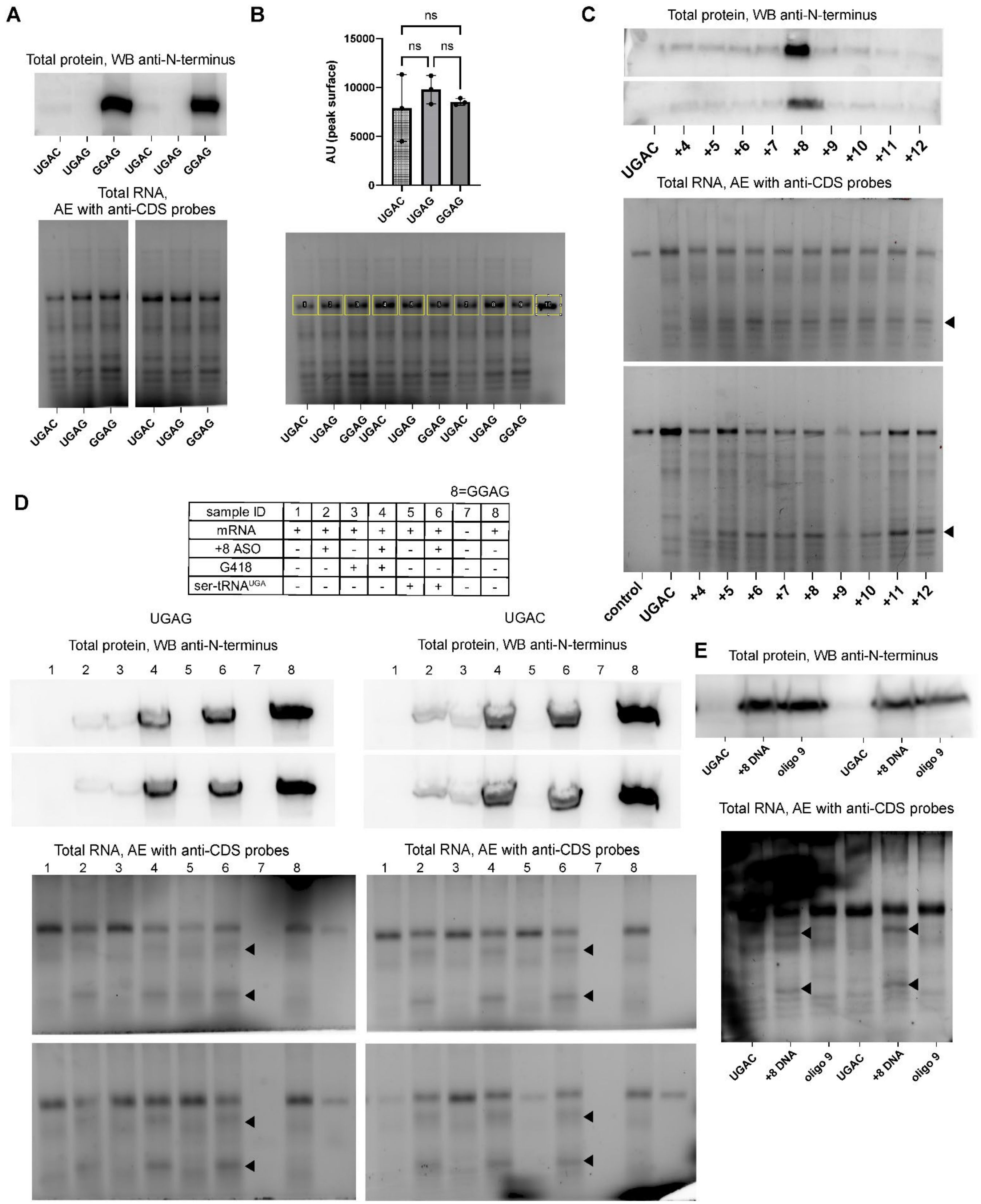

Figure S2. (A) Replicates for the gels presented in Figure 2B: (top panel) western blotting of full-length protein product (via an anti N-terminus antibody); (lower panel)

RNA gel showing mRNA levels at the end of translation reaction (agarose electrophoresis; AE with fluorescent probes complimentary to CDS). (B) Quantification of intact mRNA band intensity for the experiment presented in Figure 2B (mean ± s.d., n=3; ns – non-significant according to one-way ANOVA followed by Tukey's test for multiple comparisons); (lower panel) the areas of the gel used for intensity estimation is highlighted in yellow boxes. (C) Replicates for gels presented in Figure 2C: (top panel) western blotting of full-length protein product (via an anti N-terminus antibody); (lower panel) RNA gel showing mRNA levels at the end of translation reaction (agarose electrophoresis; AE with fluorescent probes complimentary to CDS); black arrow marks mRNA degradation product observed with DNA ASOs. Control-full-length mRNA before translation in RRL. (D) Replicates of gels presented in Figures 3A and 3B: (top panel) western blotting of full-length protein product (via an anti N-terminus antibody); (lower panel) RNA gel showing mRNA levels at the end of translation reaction (agarose electrophoresis; AE with fluorescent probes complimentary to CDS); black arrow marks mRNA degradation product observed with DNA ASOs. Control-full-length mRNA before translation in RRL. (E) Replicates for the gels presented in Figure 5C: (top panel) western blotting of full-length protein product (via an anti N-terminus antibody); (lower panel) RNA gel showing mRNA levels at the end of translation reaction (agarose electrophoresis; AE with fluorescent probes complimentary to CDS); black arrow marks mRNA degradation product observed with DNA ASOs. Control-full-length mRNA before translation in RRL.

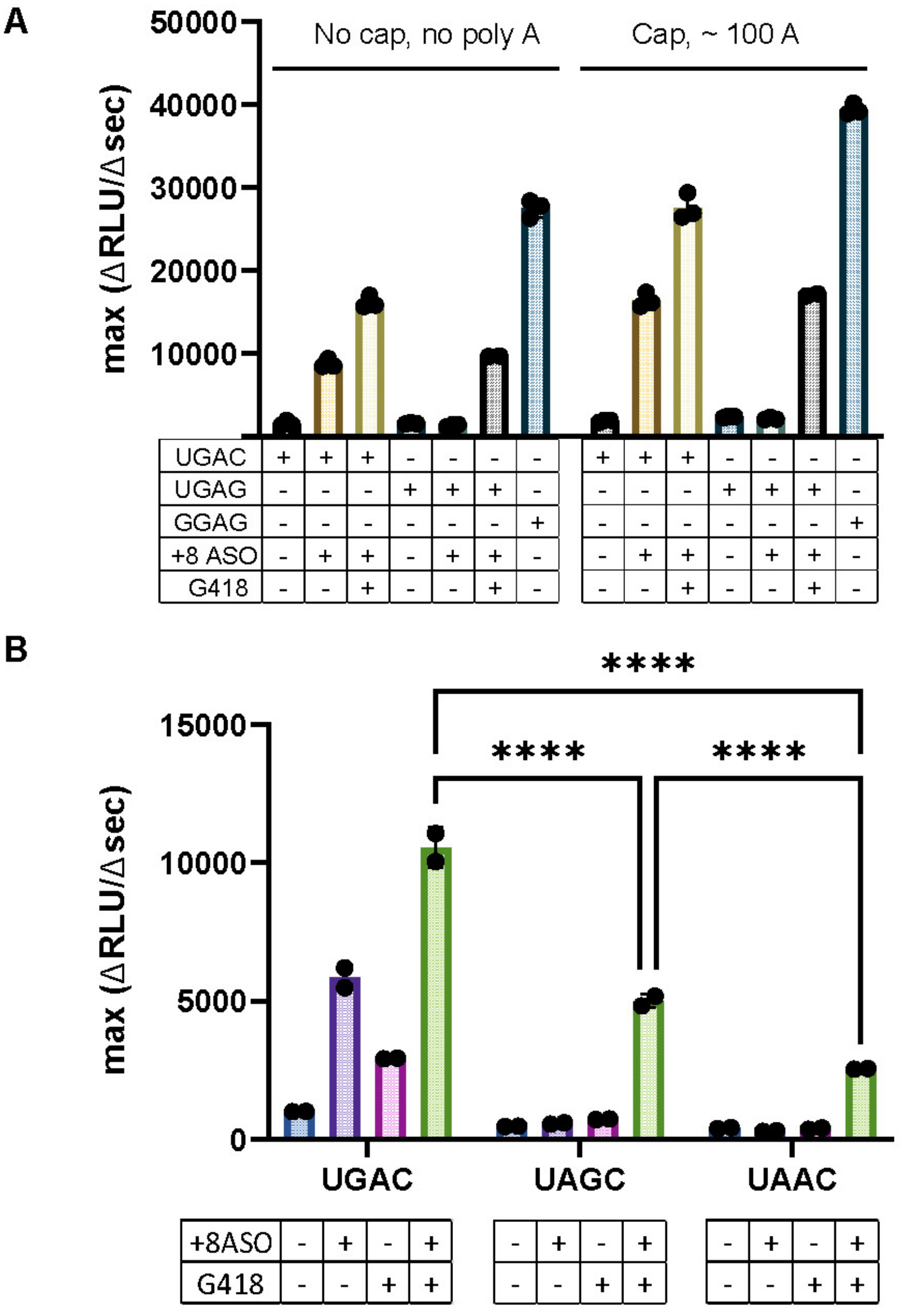

Figure S3. (A) Comparison of readthrough efficiencies in RRL for model mRNAs UGAC, UGAG and GGAG with and without m^{7}G-capping and poly(A)-tail elongation (mean ± s.d., n=3 or 2 (for UGAG+8 ASO+G418)). (B) Comparison of readthrough efficiencies in RRL for model mRNAs differing by the stop codon identity: UGA, UAG and UAA (mean ± s.d., n=2, ****=p<0.0001 according to two-way ANOVA followed by Tukey's test for multiple comparisons).

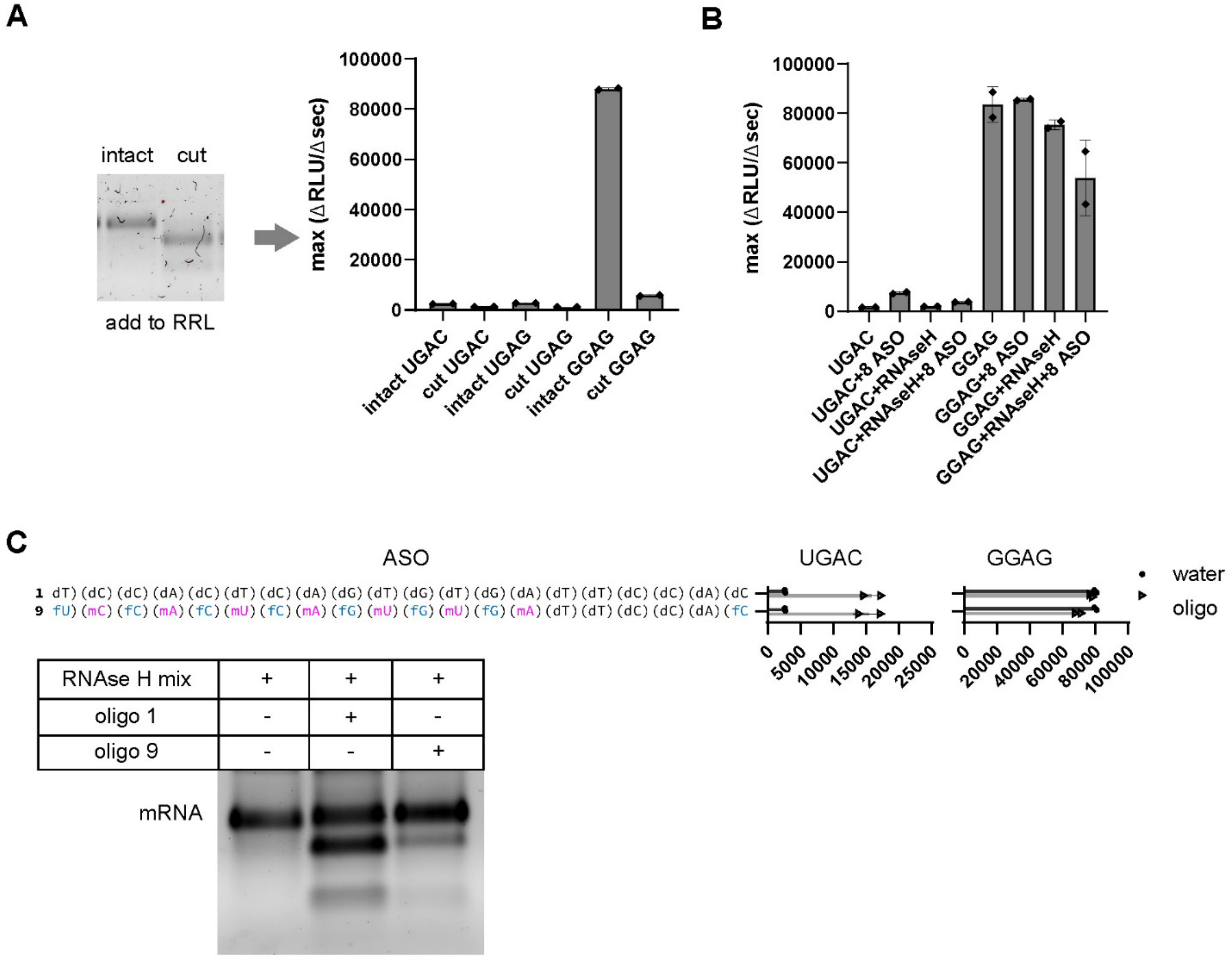

Figure S4. RNase H cleavage does not positively affect translation readthrough. (A) Translation efficiency of intact CFTR mRNAs and mRNA treated with RNase H and +8 R-ASO shown in Figure 2C (mean ± s.d., n=2). (B) Translation efficiency of intact CFTR mRNAs with and without RNase H added to RRL (mean ± s.d., n=2). (C) Chemically modified R-ASO demonstrate reduced *in vitro* RNAse H activity, partial data from Figure 5 are shown (mean ± s.d., n=2). RLU-relative luminescence units.

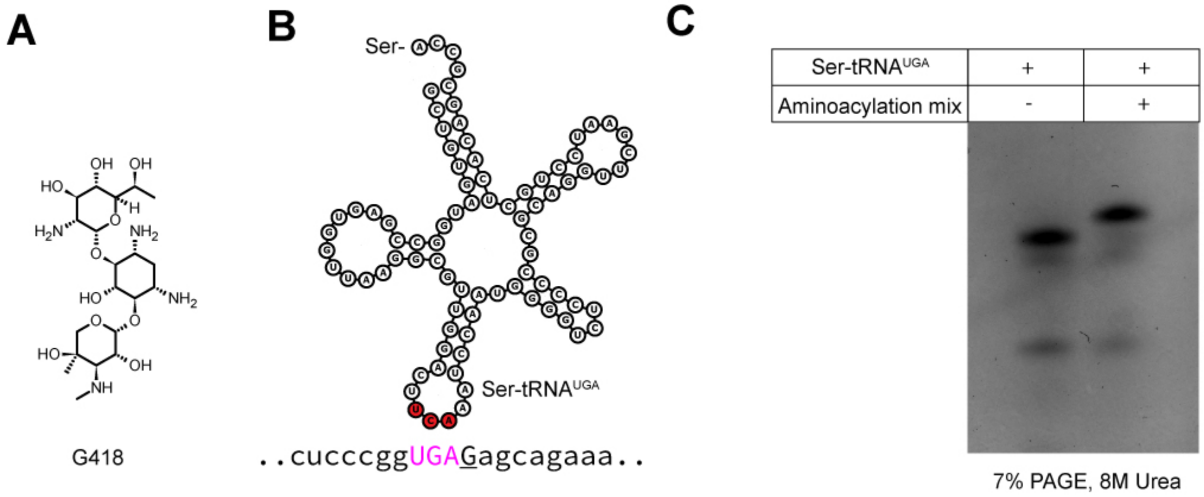

Figure S5. Readthrough-promoting agents used in this work and shown to synergize with R-ASO. (A) Chemical structure of aminoglycoside G418. (B) The cloverleaf model of suppressor ser-tRNA^{UGA}. (C) Denaturing PAGE of ser-tRNA^{UGA} before and after in vitro aminoacylation.

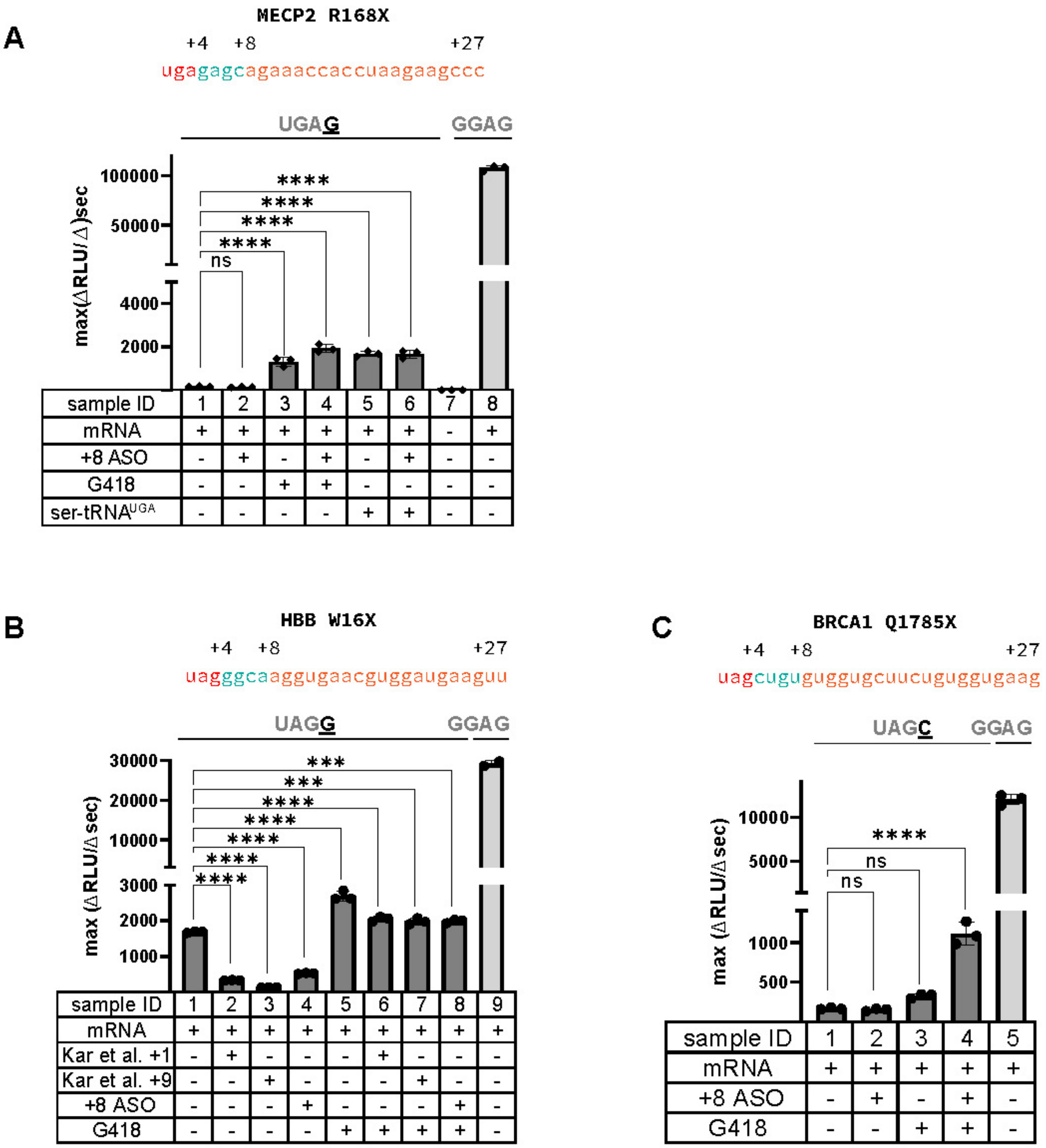

Figure S6. Action of readthrough-promoting compounds on model mRNAs encoding for: (**A**) RETT R168X nonsense mutation; (**B**) HBB W16X nonsense mutation; (**C**) BRCA1 Q1785X nonsense mutation (mean ± s.d., n=3 or n=2 (for GGAG control in A and B); ns – non-significant, ***=p<0.001, ****=p<0.0001 according to one-way ANOVA followed by Dunnetts's test for multiple comparisons).
